# 1-[2-(4-Nitro­phen­yl)-5-(5-phenyl-1,2-oxazol-3-yl)-1,2,3,4-tetra­hydro­quinolin-4-yl]pyrrolidin-2-one monohydrate

**DOI:** 10.1107/S1600536810052463

**Published:** 2010-12-18

**Authors:** Margarita Gutierrez, Gabriel Vallejos, Carlos Fernández, Alejandro Cárdenas, Iván Brito

**Affiliations:** a Instituto de Química de Recursos Naturales, Universidad de Talca, Casilla 747, Talca, Chile; b Instituto de Química, Universidad Austral de Chile, Valdivia, Chile; c Departamento de Física, Facultad de Ciencias Básicas, Universidad de Antofagasta, Casilla 170, Antofagasta, Chile; d Departamento de Química, Facultad de Ciencias Básicas, Universidad de Antofagasta, Casilla 170, Antofagasta, Chile

## Abstract

The title compound, C_28_H_24_N_4_O_4_·H_2_O, crystallizes with two organic mol­ecules and two solvent water mol­ecules in the asymmetric unit. The most obvious difference between the mol­ecules is the torsion angles between the isoxazole ring and the benzene and phenyl rings [47.0 (2)/56.4 (2) and 33.3 (2)/11.0 (2)°, respectively]. Another important difference is observed in the rotation of the nitro group with respect to the phenyl groups [3.5 (6) and 31.1 (6)°]. The pyrrolidinone fragment is *cis* oriented with respect to the 4-nitro­phenyl fragment. In the crystal, mol­ecules are linked into centrosymmetric *R*_4_^2^(8) and *R*_4_^4^(20) motifs by O—H⋯O and N—H⋯O inter­actions.

## Related literature

For pharmacological activity of quinoline, see: Shi *et al.* (2008 ▶); Lunniss *et al.* (2009 ▶); He *et al.* (2005 ▶); Eswaran *et al.* (2010 ▶). For the synthesis and medicinal uses of quinolines, see: Kalita *et al.* (2006 ▶); Kouznetsov *et al.* (2005 ▶); Sankaran *et al.* (2010 ▶). For reactions of isoxazoles see: Taldone *et al.* (2008 ▶); Narlawar *et al.* (2008 ▶); Velaparthi *et al.* (2008 ▶); Rizzi *et al.* (2008 ▶); Lautens & Roy (2000 ▶); Broggini *et al.* (2005 ▶); Kotera *et al.* (1970 ▶). For hydrogen-bond motifs, see: Bernstein *et al.* (1995 ▶).
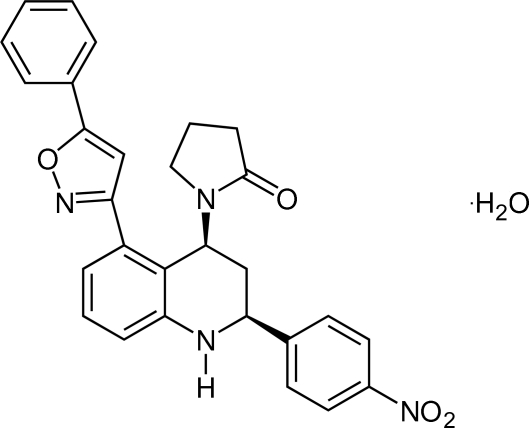


## Experimental

### 

#### Crystal data

C_28_H_24_N_4_O_4_·H_2_O
*M* *_r_* = 498.53
Triclinic, 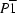

*a* = 13.516 (8) Å
*b* = 14.193 (6) Å
*c* = 14.987 (11) Å
α = 70.151 (10)°
β = 79.62 (2)°
γ = 69.700 (9)°
*V* = 2530 (3) Å^3^
*Z* = 4
Mo *K*α radiation
μ = 0.09 mm^−1^
*T* = 293 K
0.39 × 0.17 × 0.12 mm

#### Data collection

Nonius KappaCCD diffractometer
21159 measured reflections
11596 independent reflections
7891 reflections with *I* > 2σ(*I*)
*R*_int_ = 0.090

#### Refinement

*R*[*F* ^2^ > 2σ(*F* ^2^)] = 0.098
*wR*(*F* ^2^) = 0.240
*S* = 1.16
11596 reflections
691 parameters
H atoms treated by a mixture of independent and constrained refinement
Δρ_max_ = 0.34 e Å^−3^
Δρ_min_ = −0.33 e Å^−3^

### 

Data collection: *COLLECT* (Nonius, 2000 ▶); cell refinement: *DENZO-SMN* (Otwinowski & Minor, 1997 ▶); data reduction: *DENZO-SMN*; program(s) used to solve structure: *SIR97* (Altomare *et al.*, 1999 ▶); program(s) used to refine structure: *SHELXL97* (Sheldrick, 2008 ▶); molecular graphics: *ORTEP-3 for Windows* (Farrugia, 1997 ▶) and *PLATON* (Spek, 2009 ▶); software used to prepare material for publication: *WinGX* (Farrugia, 1999 ▶).

## Supplementary Material

Crystal structure: contains datablocks I, global. DOI: 10.1107/S1600536810052463/om2389sup1.cif

Structure factors: contains datablocks I. DOI: 10.1107/S1600536810052463/om2389Isup2.hkl

Additional supplementary materials:  crystallographic information; 3D view; checkCIF report

## Figures and Tables

**Table 1:** Hydrogen-bond geometry (Å, °)

*D*—H⋯*A*	*D*—H	H⋯*A*	*D*⋯*A*	*D*—H⋯*A*
O1*W*—H1*WA*⋯O4^i^	0.83 (7)	2.07 (7)	2.904 (5)	173 (6)
O1*W*—H1*WB*⋯O4^ii^	1.03 (8)	1.87 (8)	2.877 (5)	167 (6)
O2*W*—H2*WB*⋯O7	0.97 (8)	1.80 (9)	2.754 (5)	165 (8)
N6—H6*N*⋯O2*W*^iii^	0.83 (4)	2.13 (4)	2.958 (5)	179 (5)
O2*W*—H2*WA*⋯O1*W*	0.80 (6)	2.09 (6)	2.883 (6)	175 (6)

Symmetry codes: (i) 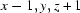; (ii) 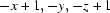; (iii) 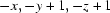.

**Fig. 1.**
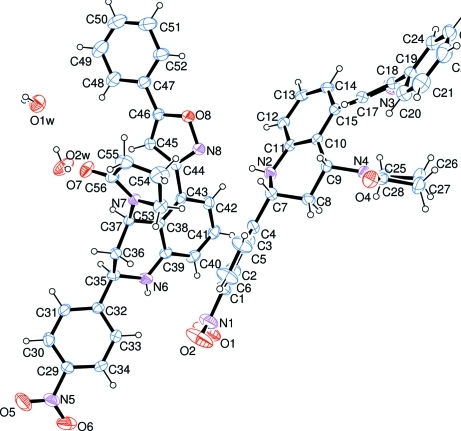
The structure of the title compound showing the atom-numbering scheme. Displacement ellipsoids are plotted at the 30% probability level.

**Fig. 2.**
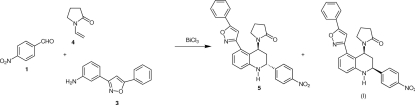
Synthesis scheme of the title compound.

## supplementary crystallographic information

### Comment

Nitrogen containing heterocycles are indispensable structural units for medicinal chemists (Sankaran *et al.*, 2010). Compounds possessing the quinoline system have wide applications as drugs and pharmaceuticals and also occur as structural frameworks in natural products (Kalita *et al.*, 2006). They also have several pharmacological activities such as anti-breast cancer (Shi *et al.*, 2008), selective PDE4 inhibition (Lunniss *et al.*, 2009), immuno modulatory (He *et al.*, 2005), antimycobacterial agents (Eswaran *et al.*, 2010), among others.

Quinoline and derivatives represent the major class of heterocycles, and a number of preparations have been known since the late 1800's. The quinoline skeleton is often used for the design of many synthetic compounds with diverse pharmacological properties. Several syntheses of quinolines are known, but due to their importance, the development of new synthetic approaches remains an active research area (Kouznetsov *et al.*, 2005).

The isoxazoles form a relevant group of biologically active compounds with a wide range of applications, including Hsp90 super chaperone complex inhibitors (Taldone *et al.*, 2008), tau aggregation inhibitors for treatment of Alzheimer's disease (Narlawar *et al.*, 2008), *Mycobacterium tuberculosis* pantothenate synthetase inhibitors (Velaparthi *et al.*, 2008) and neuronal nicotinic acetylcholine receptor agonist effect (Rizzi *et al.*, 2008).

A considerable number of methods to synthesize substituted isoxazoles have been published including approaches based on intramolecular cycloadditions, condensations, and intramolecular cyclizations of amino acids. These methods sometimes suffer in their versatility, convenience and yield (Lautens & Roy, 2000). The isoxazole ring can be synthesized by 1,3-dipolar cycloaddition reactions between nitrile oxide and alkyne, and that reaction may be catalyzed by copper(II). Cycloaddition reactions are among the most useful reactions in synthetic and mechanistic organic chemistry (Broggini *et al.*, 2005).

Isoxazoles have a rich chemistry because of their easy reductive cleavage and susceptibility to ring transformations (Kotera *et al.*, 1970). Depending on the substitution patterns, isoxazoles can be used as reagents for the imino-Diels-Alder condensation between anilines, aldehydes and electron-rich alkenes to generate tetrahydroquinolines with different selected substitution patterns. Due to this fact, the combination of the two heterocycles rings into a new chemical entityis of interest as no examples are known on chemical literature to date. Many molecules widely used today consist of fusions of rings; an example is the case of penicillins, where in the isoxazole ring incorporation allowed obtaining stable derivatives catalyzed degradation by gastric acid level (flucloxacillin and cloxacillin).

We report here the crystal structure of a novel synthetic derivative *cis* quinoline-isoxazole by imino Diels-Alder cycloaddition, Fig. 2. The title compound, C_28_H_24_N_4_O_4_.H_2_O, crystallizes with two organic molecules and two solvent water molecule in the asymmetric unit., Fig. 1. The most obvious difference between the molecules is the torsion angles between the isoxazole ring and the benzene and phenyl rings [47.0 (2); 56.4 (2) and 33.3 (2); 11.0 (2)°] respectively. Anther important difference is observed in the rotation of the nitro group with respect to the phenyl group [3.5 (6)°; 31.1 (6)°]. The pyrrolidinone fragment is *cis* oriented with respect to the 4-nitrophenyl fragment. In the crystal the molecules are linked into centrosymmetric *R*^2^_4_(8) and *R*^4^_4_(20) motifs by O—H···O and N—H···O interactions, (Bernstein *et al.*, 1995). There are six intramolecular hydrogen bonds which stabilized the molecular conformation in both molecules, Table 1.

### Experimental

A mixture of 3-(3-aminophenyl)-5-phenylisoxazole (2.8 mmol) 3 and 4-nitrobenzaldehyde (3.4 mmol) 1 in anhydrous CH_3_CN (15 ml) was stirred at room temperature for 30 min. BiCl_3_ (20 mol%) was added. Over a period of 20 min, a solution the *N*-vinyl-2-pyrrolidone (NVP) (5.5 mmol) 4 in CH_3_CN (10 ml) was added dropwise. The resulting mixture was stirred for 10–14 h. After completion of the reaction as indicated by TLC, the reaction mixture was diluted with water (30 ml) and extracted with ethyl acetate (3× 15 ml). The organic layer was separated and dried (Na_2_SO_4_), concentrated in vacuum and the resulting product was purified by column chromatography (silica gel) using PE and EtOAc mixtures. Results for derivatives *trans* and *cis* quinoline-isoxazole 5 and the title compound, see Figure 2. Solid crystalline mp 215–217 °C. The crystals were obtaned by slow evaporation of a solution of the title compound in a THF:H_2_O (1:1v/v) mixture. RMN-^1^H(CDCl_3_), 400 MHz, δ): 8.14 (2*H*, d, *J* = 4.0); 7.77 (1*H*, d, *J*= 8.0); 7.59 (2*H*, d, *J* = 8.0); 7.42(2*H*, d, *J* = 8.0); 7.17 (1*H*, t, *J* = 8.0); 6.93 (1*H*, s); 6.86 (2*H*, dd, *J* = 8.0 and 2.0); 6.80 (1*H*, d, *J* = 8.0); 6.65 (1*H*, s); 4.59 (1*H*, d, J = 12.0 and 1.0); 4.51 (1*H*, br.s); 4.41 (1*H*, s); 2.93 (2*H*, m); 1.98 (2*H*, m); 1.71 (2*H*, m), 1.57 (2*H*, m). RMN-^13^H(CDCl_3_), 400 MHz,?d): 174.58, 168.95, 162.92, 149.97,147.26, 146.71, 130.03, 129.61, 128.87, 128.54, 127.13, 127.08, 127.08, 125.82,123.74, 117.35, 116.58, 100.24, 54.76, 46.93, 42.32, 34.92, 30.46, 17.25. MS *m/z* (EI): 480. Anal. Calcd. for C_28_H_24_N_4_O_4_: C, 69.99;H,5.03; N, 11.66. Found: C, 69.92; H, 5.05; N, 11.79.

### Refinement

The positions of the O1W, O2W, N2 and N6 H atoms were refined freely along with isotropic displacement parameters. All other H atoms were placed in geometrically idealized positions (C—H = 0.93–0.98 Å) and constrained to ride on their parent atoms with *U*_iso_(H) = 1.2*U*_eq_(C).

### Crystal data

**Table:**

C_28_H_24_N_4_O_4_·H_2_O	*Z* = 4
*M**_r_* = 498.53	*F*(000) = 1048
Triclinic, *P*1	*D*_x_ = 1.309 Mg m^−^^3^
Hall symbol: -P 1	Mo *K*α radiation, λ = 0.71073 Å
*a* = 13.516 (8) Å	Cell parameters from 7466 reflections
*b* = 14.193 (6) Å	θ = 1.6–27.7°
*c* = 14.987 (11) Å	µ = 0.09 mm^−^^1^
α = 70.151 (10)°	*T* = 293 K
β = 79.62 (2)°	Prism, yellow
γ = 69.700 (9)°	0.39 × 0.17 × 0.12 mm
*V* = 2530 (3) Å^3^	

### Data collection

**Table:**

Nonius KappaCCD diffractometer	7891 reflections with *I* > 2σ(*I*)
Radiation source: fine-focus sealed tube	*R*_int_ = 0.090
graphite	θ_max_ = 27.7°, θ_min_ = 1.6°
φ and ω scans with κ offsets	*h* = 0→17
21159 measured reflections	*k* = −16→18
11596 independent reflections	*l* = −18→19

### Refinement

**Table:**

Refinement on *F*^2^	Primary atom site location: structure-invariant direct methods
Least-squares matrix: full	Secondary atom site location: difference Fourier map
*R*[*F*^2^ > 2σ(*F*^2^)] = 0.098	Hydrogen site location: inferred from neighbouring sites
*wR*(*F*^2^) = 0.240	H atoms treated by a mixture of independent and constrained refinement
*S* = 1.16	*w* = 1/[σ^2^(*F*_o_^2^) + (0.0836*P*)^2^ + 2.2406*P*] where *P* = (*F*_o_^2^ + 2*F*_c_^2^)/3
11596 reflections	(Δ/σ)_max_ < 0.001
691 parameters	Δρ_max_ = 0.34 e Å^−^^3^
0 restraints	Δρ_min_ = −0.33 e Å^−^^3^

### Special details

**Table:**

Geometry. All s.u.'s (except the s.u. in the dihedral angle between two l.s. planes) are estimated using the full covariance matrix. The cell s.u.'s are taken into account individually in the estimation of s.u.'s in distances, angles and torsion angles; correlations between s.u.'s in cell parameters are only used when they are defined by crystal symmetry. An approximate (isotropic) treatment of cell s.u.'s is used for estimating s.u.'s involving l.s. planes.
Refinement. Refinement of *F*^2^ against ALL reflections. The weighted *R*-factor *wR* and goodness of fit *S* are based on *F*^2^, conventional *R*-factors *R* are based on *F*, with *F* set to zero for negative *F*^2^. The threshold expression of *F*^2^ > 2σ(*F*^2^) is used only for calculating *R*-factors(gt) *etc*. and is not relevant to the choice of reflections for refinement. *R*-factors based on *F*^2^ are statistically about twice as large as those based on *F*, and *R*- factors based on ALL data will be even larger.

### Fractional atomic coordinates and isotropic or equivalent isotropic displacement parameters (Å^2^)

**Table:**

	*x*	*y*	*z*	*U*_iso_*/*U*_eq_	
O1	0.1893 (4)	0.5828 (4)	0.0899 (4)	0.151 (2)	
O2	0.2061 (4)	0.4662 (4)	0.0265 (4)	0.1459 (19)	
O3	1.18855 (17)	0.0338 (2)	0.28972 (19)	0.0595 (7)	
O4	0.9406 (2)	0.1193 (2)	0.07318 (17)	0.0621 (7)	
O5	−0.4045 (3)	0.5670 (3)	0.1648 (3)	0.1073 (13)	
O6	−0.3530 (3)	0.7007 (3)	0.0855 (3)	0.0982 (11)	
O7	0.1570 (2)	0.12322 (19)	0.5951 (2)	0.0675 (7)	
O8	0.49271 (17)	0.14888 (18)	0.61723 (17)	0.0540 (6)	
N1	0.2355 (3)	0.4985 (3)	0.0794 (3)	0.0850 (11)	
N2	0.6331 (2)	0.2718 (2)	0.3431 (2)	0.0473 (7)	
H2N	0.589 (3)	0.264 (3)	0.388 (3)	0.077 (14)*	
N3	1.1088 (2)	0.1043 (2)	0.3322 (2)	0.0582 (8)	
N4	0.90577 (19)	0.22917 (19)	0.16467 (17)	0.0405 (6)	
N5	−0.3424 (3)	0.6164 (3)	0.1471 (3)	0.0681 (9)	
N6	0.0703 (2)	0.5391 (2)	0.3501 (2)	0.0548 (8)	
H6N	0.039 (3)	0.603 (3)	0.331 (3)	0.060 (11)*	
N7	0.2327 (2)	0.21999 (18)	0.46358 (18)	0.0399 (6)	
N8	0.4633 (2)	0.2353 (2)	0.5345 (2)	0.0511 (7)	
C1	0.3325 (3)	0.4336 (3)	0.1285 (3)	0.0596 (9)	
C2	0.3650 (3)	0.4683 (3)	0.1888 (3)	0.0616 (10)	
H2	0.3254	0.5318	0.2002	0.074*	
C3	0.4581 (3)	0.4079 (3)	0.2332 (3)	0.0538 (9)	
H3	0.4811	0.4315	0.2742	0.065*	
C4	0.5168 (2)	0.3135 (3)	0.2172 (2)	0.0447 (7)	
C5	0.4787 (3)	0.2782 (4)	0.1591 (3)	0.0767 (13)	
H5	0.5159	0.2128	0.1505	0.092*	
C6	0.3876 (4)	0.3372 (4)	0.1137 (4)	0.0863 (15)	
H6	0.3633	0.3129	0.0741	0.104*	
C7	0.6221 (2)	0.2472 (3)	0.2599 (2)	0.0445 (7)	
H7	0.6272	0.1726	0.2780	0.053*	
C8	0.7137 (2)	0.2665 (3)	0.1879 (2)	0.0460 (8)	
H8A	0.7118	0.3392	0.1728	0.055*	
H8B	0.7061	0.2548	0.1297	0.055*	
C9	0.8203 (2)	0.1933 (2)	0.2269 (2)	0.0382 (7)	
H9	0.8288	0.1225	0.2253	0.046*	
C10	0.8255 (2)	0.1874 (2)	0.3297 (2)	0.0352 (6)	
C11	0.7322 (2)	0.2302 (2)	0.3808 (2)	0.0374 (7)	
C12	0.7374 (3)	0.2340 (2)	0.4721 (2)	0.0438 (7)	
H12	0.6758	0.2628	0.5055	0.053*	
C13	0.8327 (3)	0.1955 (2)	0.5127 (2)	0.0464 (8)	
H13	0.8354	0.2013	0.5721	0.056*	
C14	0.9243 (3)	0.1482 (2)	0.4654 (2)	0.0452 (7)	
H14	0.9881	0.1197	0.4941	0.054*	
C15	0.9209 (2)	0.1434 (2)	0.3746 (2)	0.0380 (7)	
C16	1.0207 (2)	0.0866 (2)	0.3289 (2)	0.0402 (7)	
C17	1.0388 (2)	0.0063 (2)	0.2874 (2)	0.0407 (7)	
H17	0.9888	−0.0202	0.2779	0.049*	
C18	1.1431 (2)	−0.0240 (2)	0.2643 (2)	0.0429 (7)	
C19	1.2137 (2)	−0.1041 (2)	0.2210 (2)	0.0470 (8)	
C20	1.1780 (3)	−0.1278 (3)	0.1532 (3)	0.0616 (10)	
H20	1.1094	−0.0925	0.1346	0.074*	
C21	1.2446 (4)	−0.2045 (3)	0.1127 (3)	0.0763 (12)	
H21	1.2205	−0.2210	0.0673	0.092*	
C22	1.3470 (4)	−0.2560 (3)	0.1400 (4)	0.0783 (13)	
H22	1.3920	−0.3065	0.1123	0.094*	
C23	1.3819 (3)	−0.2328 (3)	0.2078 (3)	0.0695 (12)	
H23	1.4508	−0.2678	0.2259	0.083*	
C24	1.3166 (3)	−0.1586 (3)	0.2491 (3)	0.0558 (9)	
H24	1.3407	−0.1443	0.2959	0.067*	
C25	0.9335 (3)	0.3197 (3)	0.1655 (3)	0.0564 (9)	
H25A	0.9496	0.3119	0.2287	0.068*	
H25B	0.8764	0.3847	0.1444	0.068*	
C26	1.0301 (4)	0.3184 (4)	0.0966 (4)	0.0884 (15)	
H26A	1.0248	0.3888	0.0546	0.106*	
H26B	1.0934	0.2921	0.1308	0.106*	
C27	1.0343 (3)	0.2479 (4)	0.0412 (3)	0.0712 (12)	
H27A	1.1048	0.1988	0.0386	0.085*	
H27B	1.0150	0.2886	−0.0232	0.085*	
C28	0.9561 (3)	0.1901 (3)	0.0928 (2)	0.0475 (8)	
C29	−0.2518 (2)	0.5753 (3)	0.2061 (2)	0.0485 (8)	
C30	−0.2560 (3)	0.5056 (3)	0.2951 (3)	0.0528 (9)	
H30	−0.3141	0.4813	0.3177	0.063*	
C31	−0.1722 (3)	0.4724 (2)	0.3505 (2)	0.0483 (8)	
H31	−0.1750	0.4265	0.4117	0.058*	
C32	−0.0837 (2)	0.5060 (2)	0.3171 (2)	0.0404 (7)	
C33	−0.0809 (3)	0.5730 (3)	0.2250 (3)	0.0583 (9)	
H33	−0.0210	0.5939	0.2002	0.070*	
C34	−0.1656 (3)	0.6091 (3)	0.1698 (3)	0.0630 (10)	
H34	−0.1640	0.6556	0.1088	0.076*	
C35	0.0048 (2)	0.4706 (2)	0.3819 (2)	0.0433 (7)	
H35	−0.0272	0.4730	0.4455	0.052*	
C36	0.0762 (2)	0.3586 (2)	0.3899 (2)	0.0422 (7)	
H36A	0.1121	0.3552	0.3284	0.051*	
H36B	0.0338	0.3111	0.4095	0.051*	
C37	0.1584 (2)	0.3241 (2)	0.4628 (2)	0.0368 (7)	
H37	0.1205	0.3172	0.5258	0.044*	
C38	0.2140 (2)	0.4068 (2)	0.4446 (2)	0.0340 (6)	
C39	0.1663 (3)	0.5109 (2)	0.3872 (2)	0.0411 (7)	
C40	0.2179 (3)	0.5872 (2)	0.3665 (2)	0.0515 (9)	
H40	0.1864	0.6553	0.3287	0.062*	
C41	0.3136 (3)	0.5631 (3)	0.4009 (3)	0.0518 (8)	
H41	0.3474	0.6143	0.3851	0.062*	
C42	0.3605 (3)	0.4633 (3)	0.4591 (2)	0.0472 (8)	
H42	0.4249	0.4477	0.4833	0.057*	
C43	0.3107 (2)	0.3856 (2)	0.4816 (2)	0.0375 (7)	
C44	0.3630 (2)	0.2837 (2)	0.5516 (2)	0.0379 (7)	
C45	0.3240 (2)	0.2333 (2)	0.6422 (2)	0.0426 (7)	
H45	0.2557	0.2527	0.6699	0.051*	
C46	0.4071 (3)	0.1506 (2)	0.6807 (2)	0.0443 (7)	
C47	0.4233 (3)	0.0707 (3)	0.7737 (3)	0.0532 (9)	
C48	0.3381 (4)	0.0623 (4)	0.8380 (3)	0.0809 (13)	
H48	0.2702	0.1046	0.8209	0.097*	
C49	0.3530 (5)	−0.0096 (5)	0.9289 (4)	0.0997 (17)	
H49	0.2951	−0.0155	0.9720	0.120*	
C50	0.4522 (5)	−0.0710 (4)	0.9544 (4)	0.0913 (16)	
H50	0.4618	−0.1184	1.0151	0.110*	
C51	0.5376 (5)	−0.0636 (3)	0.8917 (4)	0.0815 (14)	
H51	0.6052	−0.1052	0.9098	0.098*	
C52	0.5234 (3)	0.0061 (3)	0.8010 (3)	0.0646 (10)	
H52	0.5817	0.0097	0.7578	0.077*	
C53	0.3095 (3)	0.2023 (3)	0.3841 (3)	0.0539 (9)	
H53A	0.3517	0.2498	0.3662	0.065*	
H53B	0.2747	0.2113	0.3292	0.065*	
C54	0.3765 (4)	0.0892 (3)	0.4239 (3)	0.0801 (13)	
H54A	0.3990	0.0543	0.3749	0.096*	
H54B	0.4386	0.0859	0.4503	0.096*	
C55	0.3063 (3)	0.0394 (3)	0.4998 (3)	0.0656 (11)	
H55A	0.3457	−0.0095	0.5539	0.079*	
H55B	0.2745	0.0018	0.4759	0.079*	
C56	0.2227 (3)	0.1298 (2)	0.5272 (3)	0.0484 (8)	
O1W	0.0770 (3)	0.0833 (3)	0.9068 (2)	0.0812 (9)	
H1WA	0.034 (5)	0.092 (5)	0.953 (5)	0.13 (2)*	
H1WB	0.077 (5)	0.012 (6)	0.904 (5)	0.16 (3)*	
O2W	0.0413 (3)	0.2325 (2)	0.7199 (3)	0.1003 (13)	
H2WA	0.050 (4)	0.194 (4)	0.773 (4)	0.092 (18)*	
H2WB	0.071 (6)	0.191 (6)	0.676 (6)	0.17 (3)*	

### Atomic displacement parameters (Å^2^)

**Table:**

	*U*^11^	*U*^22^	*U*^33^	*U*^12^	*U*^13^	*U*^23^
O1	0.112 (3)	0.108 (3)	0.223 (6)	0.031 (3)	−0.097 (4)	−0.061 (3)
O2	0.110 (3)	0.141 (4)	0.199 (5)	−0.002 (3)	−0.100 (3)	−0.059 (4)
O3	0.0358 (12)	0.0650 (15)	0.0817 (18)	−0.0172 (11)	0.0031 (12)	−0.0290 (14)
O4	0.0702 (17)	0.0703 (17)	0.0508 (15)	−0.0192 (13)	0.0074 (12)	−0.0327 (13)
O5	0.066 (2)	0.099 (3)	0.153 (4)	−0.0306 (19)	−0.044 (2)	−0.009 (2)
O6	0.094 (2)	0.085 (2)	0.093 (2)	−0.0120 (18)	−0.047 (2)	0.0057 (19)
O7	0.0827 (19)	0.0509 (14)	0.0628 (17)	−0.0323 (13)	0.0064 (15)	−0.0031 (12)
O8	0.0400 (12)	0.0549 (14)	0.0518 (14)	−0.0061 (10)	−0.0069 (11)	−0.0043 (11)
N1	0.064 (2)	0.083 (3)	0.102 (3)	−0.009 (2)	−0.037 (2)	−0.018 (2)
N2	0.0372 (15)	0.0636 (17)	0.0402 (15)	−0.0123 (13)	0.0027 (13)	−0.0208 (13)
N3	0.0425 (16)	0.0625 (18)	0.077 (2)	−0.0169 (14)	−0.0022 (15)	−0.0299 (16)
N4	0.0400 (14)	0.0430 (13)	0.0326 (13)	−0.0107 (11)	0.0035 (11)	−0.0093 (11)
N5	0.0507 (19)	0.063 (2)	0.078 (2)	−0.0022 (16)	−0.0216 (17)	−0.0137 (18)
N6	0.0608 (19)	0.0260 (13)	0.071 (2)	−0.0093 (13)	−0.0264 (16)	0.0001 (13)
N7	0.0441 (14)	0.0292 (12)	0.0431 (14)	−0.0093 (10)	−0.0047 (11)	−0.0081 (10)
N8	0.0393 (15)	0.0532 (16)	0.0481 (16)	−0.0086 (12)	−0.0032 (12)	−0.0052 (13)
C1	0.0437 (19)	0.066 (2)	0.068 (2)	−0.0122 (17)	−0.0178 (18)	−0.0163 (19)
C2	0.054 (2)	0.057 (2)	0.068 (3)	−0.0062 (17)	−0.0077 (19)	−0.0221 (19)
C3	0.052 (2)	0.060 (2)	0.054 (2)	−0.0148 (17)	−0.0061 (16)	−0.0252 (17)
C4	0.0364 (16)	0.0546 (19)	0.0473 (18)	−0.0152 (14)	−0.0011 (14)	−0.0204 (15)
C5	0.069 (3)	0.072 (3)	0.098 (3)	0.001 (2)	−0.033 (2)	−0.046 (2)
C6	0.077 (3)	0.087 (3)	0.112 (4)	−0.006 (2)	−0.047 (3)	−0.050 (3)
C7	0.0393 (17)	0.0489 (17)	0.0470 (18)	−0.0125 (14)	−0.0029 (14)	−0.0178 (14)
C8	0.0408 (17)	0.0587 (19)	0.0368 (17)	−0.0098 (15)	−0.0060 (13)	−0.0163 (15)
C9	0.0369 (16)	0.0419 (15)	0.0348 (16)	−0.0123 (12)	0.0016 (12)	−0.0121 (12)
C10	0.0404 (16)	0.0344 (14)	0.0307 (15)	−0.0147 (12)	−0.0011 (12)	−0.0069 (11)
C11	0.0413 (16)	0.0367 (14)	0.0335 (15)	−0.0160 (12)	0.0009 (12)	−0.0073 (12)
C12	0.0503 (19)	0.0446 (16)	0.0335 (16)	−0.0141 (14)	0.0039 (14)	−0.0121 (13)
C13	0.066 (2)	0.0429 (16)	0.0297 (16)	−0.0169 (15)	−0.0071 (15)	−0.0079 (13)
C14	0.0485 (18)	0.0425 (16)	0.0410 (18)	−0.0125 (14)	−0.0126 (14)	−0.0048 (14)
C15	0.0404 (16)	0.0351 (14)	0.0370 (16)	−0.0137 (12)	−0.0031 (13)	−0.0066 (12)
C16	0.0377 (16)	0.0418 (16)	0.0365 (16)	−0.0131 (13)	−0.0073 (13)	−0.0029 (13)
C17	0.0363 (16)	0.0426 (16)	0.0395 (17)	−0.0122 (13)	−0.0060 (13)	−0.0058 (13)
C18	0.0384 (16)	0.0425 (16)	0.0430 (18)	−0.0136 (13)	−0.0056 (13)	−0.0045 (13)
C19	0.0409 (17)	0.0422 (16)	0.0453 (19)	−0.0103 (14)	0.0038 (14)	−0.0035 (14)
C20	0.055 (2)	0.062 (2)	0.062 (2)	−0.0146 (18)	−0.0012 (18)	−0.0163 (19)
C21	0.091 (3)	0.072 (3)	0.070 (3)	−0.028 (2)	0.009 (2)	−0.030 (2)
C22	0.075 (3)	0.057 (2)	0.088 (3)	−0.014 (2)	0.025 (3)	−0.026 (2)
C23	0.046 (2)	0.058 (2)	0.079 (3)	−0.0073 (17)	0.011 (2)	−0.007 (2)
C24	0.0414 (18)	0.0536 (19)	0.058 (2)	−0.0124 (15)	0.0034 (16)	−0.0050 (16)
C25	0.064 (2)	0.0487 (19)	0.054 (2)	−0.0239 (17)	0.0065 (17)	−0.0111 (16)
C26	0.085 (3)	0.094 (3)	0.092 (3)	−0.054 (3)	0.028 (3)	−0.026 (3)
C27	0.066 (3)	0.082 (3)	0.055 (2)	−0.028 (2)	0.0201 (19)	−0.014 (2)
C28	0.0420 (17)	0.0532 (19)	0.0345 (17)	−0.0055 (14)	−0.0004 (14)	−0.0080 (14)
C29	0.0374 (17)	0.0462 (17)	0.053 (2)	−0.0043 (14)	−0.0078 (15)	−0.0109 (15)
C30	0.0391 (18)	0.0516 (19)	0.063 (2)	−0.0141 (15)	0.0049 (16)	−0.0160 (17)
C31	0.0485 (19)	0.0426 (17)	0.0433 (18)	−0.0126 (14)	−0.0006 (15)	−0.0028 (14)
C32	0.0384 (16)	0.0337 (14)	0.0409 (17)	−0.0032 (12)	−0.0028 (13)	−0.0091 (12)
C33	0.0453 (19)	0.072 (2)	0.047 (2)	−0.0236 (17)	−0.0020 (16)	0.0013 (17)
C34	0.058 (2)	0.071 (2)	0.041 (2)	−0.0227 (19)	−0.0103 (17)	0.0115 (17)
C35	0.0453 (18)	0.0355 (15)	0.0422 (17)	−0.0048 (13)	−0.0077 (14)	−0.0083 (13)
C36	0.0415 (17)	0.0330 (14)	0.0496 (19)	−0.0109 (12)	−0.0063 (14)	−0.0084 (13)
C37	0.0369 (15)	0.0288 (13)	0.0393 (16)	−0.0077 (11)	0.0008 (12)	−0.0080 (12)
C38	0.0372 (15)	0.0289 (13)	0.0346 (15)	−0.0093 (11)	0.0011 (12)	−0.0107 (11)
C39	0.0509 (18)	0.0309 (14)	0.0405 (17)	−0.0116 (13)	−0.0046 (14)	−0.0098 (12)
C40	0.073 (2)	0.0301 (15)	0.049 (2)	−0.0182 (15)	−0.0061 (17)	−0.0053 (13)
C41	0.066 (2)	0.0447 (18)	0.054 (2)	−0.0321 (17)	0.0001 (17)	−0.0122 (15)
C42	0.0481 (18)	0.0510 (18)	0.0490 (19)	−0.0236 (15)	−0.0006 (15)	−0.0159 (15)
C43	0.0403 (16)	0.0349 (14)	0.0363 (16)	−0.0123 (12)	0.0033 (13)	−0.0119 (12)
C44	0.0344 (15)	0.0398 (15)	0.0414 (17)	−0.0125 (12)	−0.0012 (13)	−0.0139 (13)
C45	0.0382 (16)	0.0457 (17)	0.0423 (17)	−0.0137 (13)	−0.0010 (13)	−0.0113 (14)
C46	0.0464 (18)	0.0449 (17)	0.0445 (18)	−0.0192 (14)	−0.0052 (14)	−0.0109 (14)
C47	0.063 (2)	0.0492 (18)	0.049 (2)	−0.0230 (17)	−0.0108 (17)	−0.0072 (15)
C48	0.076 (3)	0.083 (3)	0.064 (3)	−0.026 (2)	0.003 (2)	0.001 (2)
C49	0.111 (4)	0.105 (4)	0.063 (3)	−0.044 (3)	0.009 (3)	0.003 (3)
C50	0.135 (5)	0.076 (3)	0.058 (3)	−0.040 (3)	−0.026 (3)	0.004 (2)
C51	0.108 (4)	0.060 (2)	0.077 (3)	−0.029 (2)	−0.042 (3)	0.000 (2)
C52	0.077 (3)	0.050 (2)	0.065 (2)	−0.0218 (19)	−0.024 (2)	−0.0033 (18)
C53	0.057 (2)	0.0459 (18)	0.056 (2)	−0.0086 (15)	0.0027 (17)	−0.0228 (16)
C54	0.081 (3)	0.049 (2)	0.092 (3)	0.007 (2)	−0.002 (3)	−0.030 (2)
C55	0.075 (3)	0.0348 (17)	0.082 (3)	−0.0026 (17)	−0.024 (2)	−0.0159 (18)
C56	0.059 (2)	0.0346 (16)	0.053 (2)	−0.0185 (15)	−0.0155 (17)	−0.0050 (14)
O1W	0.082 (2)	0.093 (2)	0.066 (2)	−0.0288 (18)	0.0151 (17)	−0.0289 (17)
O2W	0.129 (3)	0.0466 (16)	0.083 (3)	−0.0045 (17)	0.016 (2)	−0.0025 (17)

### Geometric parameters (Å, °)

**Table:**

O1—N1	1.191 (5)	C23—H23	0.9300
O2—N1	1.217 (6)	C24—H24	0.9300
O3—C18	1.357 (4)	C25—C26	1.511 (5)
O3—N3	1.416 (4)	C25—H25A	0.9700
O4—C28	1.228 (4)	C25—H25B	0.9700
O5—N5	1.212 (5)	C26—C27	1.483 (7)
O6—N5	1.217 (5)	C26—H26A	0.9700
O7—C56	1.225 (4)	C26—H26B	0.9700
O8—C46	1.358 (4)	C27—C28	1.505 (5)
O8—N8	1.416 (4)	C27—H27A	0.9700
N1—C1	1.469 (5)	C27—H27B	0.9700
N2—C11	1.399 (4)	C29—C34	1.365 (5)
N2—C7	1.449 (4)	C29—C30	1.370 (5)
N2—H2N	0.82 (4)	C30—C31	1.377 (5)
N3—C16	1.311 (4)	C30—H30	0.9300
N4—C28	1.345 (4)	C31—C32	1.388 (5)
N4—C25	1.463 (4)	C31—H31	0.9300
N4—C9	1.470 (4)	C32—C33	1.387 (5)
N5—C29	1.472 (5)	C32—C35	1.515 (5)
N6—C39	1.378 (4)	C33—C34	1.381 (5)
N6—C35	1.446 (4)	C33—H33	0.9300
N6—H6N	0.83 (4)	C34—H34	0.9300
N7—C56	1.346 (4)	C35—C36	1.523 (4)
N7—C53	1.461 (4)	C35—H35	0.9800
N7—C37	1.468 (3)	C36—C37	1.538 (4)
N8—C44	1.312 (4)	C36—H36A	0.9700
C1—C2	1.360 (5)	C36—H36B	0.9700
C1—C6	1.384 (6)	C37—C38	1.529 (4)
C2—C3	1.388 (5)	C37—H37	0.9800
C2—H2	0.9300	C38—C43	1.407 (4)
C3—C4	1.376 (5)	C38—C39	1.420 (4)
C3—H3	0.9300	C39—C40	1.404 (5)
C4—C5	1.382 (5)	C40—C41	1.368 (5)
C4—C7	1.519 (5)	C40—H40	0.9300
C5—C6	1.372 (6)	C41—C42	1.380 (5)
C5—H5	0.9300	C41—H41	0.9300
C6—H6	0.9300	C42—C43	1.401 (4)
C7—C8	1.526 (4)	C42—H42	0.9300
C7—H7	0.9800	C43—C44	1.490 (4)
C8—C9	1.533 (4)	C44—C45	1.407 (4)
C8—H8A	0.9700	C45—C46	1.352 (4)
C8—H8B	0.9700	C45—H45	0.9300
C9—C10	1.527 (4)	C46—C47	1.464 (5)
C9—H9	0.9800	C47—C48	1.373 (6)
C10—C15	1.405 (4)	C47—C52	1.386 (5)
C10—C11	1.409 (4)	C48—C49	1.396 (7)
C11—C12	1.402 (4)	C48—H48	0.9300
C12—C13	1.376 (5)	C49—C50	1.360 (8)
C12—H12	0.9300	C49—H49	0.9300
C13—C14	1.385 (5)	C50—C51	1.360 (7)
C13—H13	0.9300	C50—H50	0.9300
C14—C15	1.395 (5)	C51—C52	1.383 (6)
C14—H14	0.9300	C51—H51	0.9300
C15—C16	1.493 (4)	C52—H52	0.9300
C16—C17	1.407 (4)	C53—C54	1.512 (5)
C17—C18	1.340 (4)	C53—H53A	0.9700
C17—H17	0.9300	C53—H53B	0.9700
C18—C19	1.466 (5)	C54—C55	1.492 (6)
C19—C20	1.377 (5)	C54—H54A	0.9700
C19—C24	1.398 (5)	C54—H54B	0.9700
C20—C21	1.392 (6)	C55—C56	1.509 (5)
C20—H20	0.9300	C55—H55A	0.9700
C21—C22	1.384 (7)	C55—H55B	0.9700
C21—H21	0.9300	O1W—H1WA	0.83 (7)
C22—C23	1.368 (7)	O1W—H1WB	1.02 (8)
C22—H22	0.9300	O2W—H2WA	0.80 (6)
C23—C24	1.369 (6)	O2W—H2WB	0.98 (8)
			
C18—O3—N3	108.5 (2)	H26A—C26—H26B	108.6
C46—O8—N8	108.4 (2)	C26—C27—C28	105.7 (3)
O1—N1—O2	121.6 (4)	C26—C27—H27A	110.6
O1—N1—C1	119.3 (4)	C28—C27—H27A	110.6
O2—N1—C1	119.0 (4)	C26—C27—H27B	110.6
C11—N2—C7	118.4 (3)	C28—C27—H27B	110.6
C11—N2—H2N	107 (3)	H27A—C27—H27B	108.7
C7—N2—H2N	117 (3)	O4—C28—N4	125.6 (3)
C16—N3—O3	105.1 (3)	O4—C28—C27	126.2 (3)
C28—N4—C25	113.5 (3)	N4—C28—C27	108.2 (3)
C28—N4—C9	122.3 (3)	C34—C29—C30	122.0 (3)
C25—N4—C9	123.6 (2)	C34—C29—N5	118.2 (3)
O5—N5—O6	123.6 (4)	C30—C29—N5	119.8 (3)
O5—N5—C29	118.5 (3)	C29—C30—C31	118.5 (3)
O6—N5—C29	117.8 (4)	C29—C30—H30	120.7
C39—N6—C35	121.3 (3)	C31—C30—H30	120.7
C39—N6—H6N	116 (3)	C30—C31—C32	121.5 (3)
C35—N6—H6N	117 (3)	C30—C31—H31	119.3
C56—N7—C53	112.7 (3)	C32—C31—H31	119.3
C56—N7—C37	123.1 (3)	C33—C32—C31	118.0 (3)
C53—N7—C37	122.9 (2)	C33—C32—C35	122.4 (3)
C44—N8—O8	105.4 (2)	C31—C32—C35	119.6 (3)
C2—C1—C6	121.4 (4)	C34—C33—C32	121.0 (3)
C2—C1—N1	119.5 (4)	C34—C33—H33	119.5
C6—C1—N1	119.1 (4)	C32—C33—H33	119.5
C1—C2—C3	119.3 (3)	C29—C34—C33	118.9 (3)
C1—C2—H2	120.4	C29—C34—H34	120.5
C3—C2—H2	120.4	C33—C34—H34	120.5
C4—C3—C2	120.7 (3)	N6—C35—C32	111.8 (3)
C4—C3—H3	119.7	N6—C35—C36	107.9 (3)
C2—C3—H3	119.7	C32—C35—C36	112.7 (3)
C3—C4—C5	118.5 (3)	N6—C35—H35	108.1
C3—C4—C7	122.8 (3)	C32—C35—H35	108.1
C5—C4—C7	118.7 (3)	C36—C35—H35	108.1
C6—C5—C4	121.7 (4)	C35—C36—C37	110.2 (3)
C6—C5—H5	119.1	C35—C36—H36A	109.6
C4—C5—H5	119.1	C37—C36—H36A	109.6
C5—C6—C1	118.3 (4)	C35—C36—H36B	109.6
C5—C6—H6	120.9	C37—C36—H36B	109.6
C1—C6—H6	120.9	H36A—C36—H36B	108.1
N2—C7—C4	111.8 (3)	N7—C37—C38	112.6 (2)
N2—C7—C8	107.4 (3)	N7—C37—C36	109.7 (2)
C4—C7—C8	110.6 (3)	C38—C37—C36	111.5 (2)
N2—C7—H7	109.0	N7—C37—H37	107.6
C4—C7—H7	109.0	C38—C37—H37	107.6
C8—C7—H7	109.0	C36—C37—H37	107.6
C7—C8—C9	111.2 (3)	C43—C38—C39	117.7 (3)
C7—C8—H8A	109.4	C43—C38—C37	123.6 (2)
C9—C8—H8A	109.4	C39—C38—C37	118.7 (3)
C7—C8—H8B	109.4	N6—C39—C40	118.8 (3)
C9—C8—H8B	109.4	N6—C39—C38	121.5 (3)
H8A—C8—H8B	108.0	C40—C39—C38	119.7 (3)
N4—C9—C10	111.4 (2)	C41—C40—C39	121.1 (3)
N4—C9—C8	109.1 (2)	C41—C40—H40	119.4
C10—C9—C8	111.8 (2)	C39—C40—H40	119.4
N4—C9—H9	108.1	C40—C41—C42	120.4 (3)
C10—C9—H9	108.1	C40—C41—H41	119.8
C8—C9—H9	108.1	C42—C41—H41	119.8
C15—C10—C11	118.4 (3)	C41—C42—C43	119.9 (3)
C15—C10—C9	122.4 (3)	C41—C42—H42	120.1
C11—C10—C9	119.1 (3)	C43—C42—H42	120.1
N2—C11—C12	118.0 (3)	C42—C43—C38	121.2 (3)
N2—C11—C10	122.3 (3)	C42—C43—C44	115.5 (3)
C12—C11—C10	119.7 (3)	C38—C43—C44	123.2 (3)
C13—C12—C11	120.8 (3)	N8—C44—C45	111.6 (3)
C13—C12—H12	119.6	N8—C44—C43	118.7 (3)
C11—C12—H12	119.6	C45—C44—C43	129.3 (3)
C12—C13—C14	120.2 (3)	C46—C45—C44	105.4 (3)
C12—C13—H13	119.9	C46—C45—H45	127.3
C14—C13—H13	119.9	C44—C45—H45	127.3
C13—C14—C15	119.9 (3)	C45—C46—O8	109.1 (3)
C13—C14—H14	120.1	C45—C46—C47	134.1 (3)
C15—C14—H14	120.1	O8—C46—C47	116.7 (3)
C14—C15—C10	120.8 (3)	C48—C47—C52	118.6 (4)
C14—C15—C16	117.8 (3)	C48—C47—C46	119.5 (4)
C10—C15—C16	121.4 (3)	C52—C47—C46	121.8 (3)
N3—C16—C17	111.6 (3)	C47—C48—C49	120.1 (5)
N3—C16—C15	118.9 (3)	C47—C48—H48	119.9
C17—C16—C15	129.2 (3)	C49—C48—H48	119.9
C18—C17—C16	105.6 (3)	C50—C49—C48	120.1 (5)
C18—C17—H17	127.2	C50—C49—H49	120.0
C16—C17—H17	127.2	C48—C49—H49	120.0
C17—C18—O3	109.2 (3)	C51—C50—C49	120.6 (4)
C17—C18—C19	133.9 (3)	C51—C50—H50	119.7
O3—C18—C19	116.8 (3)	C49—C50—H50	119.7
C20—C19—C24	119.4 (3)	C50—C51—C52	119.8 (5)
C20—C19—C18	119.9 (3)	C50—C51—H51	120.1
C24—C19—C18	120.7 (3)	C52—C51—H51	120.1
C19—C20—C21	120.0 (4)	C51—C52—C47	120.8 (4)
C19—C20—H20	120.0	C51—C52—H52	119.6
C21—C20—H20	120.0	C47—C52—H52	119.6
C22—C21—C20	119.8 (4)	N7—C53—C54	102.8 (3)
C22—C21—H21	120.1	N7—C53—H53A	111.2
C20—C21—H21	120.1	C54—C53—H53A	111.2
C23—C22—C21	120.0 (4)	N7—C53—H53B	111.2
C23—C22—H22	120.0	C54—C53—H53B	111.2
C21—C22—H22	120.0	H53A—C53—H53B	109.1
C22—C23—C24	120.6 (4)	C55—C54—C53	104.9 (3)
C22—C23—H23	119.7	C55—C54—H54A	110.8
C24—C23—H23	119.7	C53—C54—H54A	110.8
C23—C24—C19	120.1 (4)	C55—C54—H54B	110.8
C23—C24—H24	119.9	C53—C54—H54B	110.8
C19—C24—H24	119.9	H54A—C54—H54B	108.8
N4—C25—C26	103.4 (3)	C54—C55—C56	105.0 (3)
N4—C25—H25A	111.1	C54—C55—H55A	110.8
C26—C25—H25A	111.1	C56—C55—H55A	110.8
N4—C25—H25B	111.1	C54—C55—H55B	110.8
C26—C25—H25B	111.1	C56—C55—H55B	110.8
H25A—C25—H25B	109.0	H55A—C55—H55B	108.8
C27—C26—C25	106.8 (3)	O7—C56—N7	125.6 (3)
C27—C26—H26A	110.4	O7—C56—C55	126.5 (3)
C25—C26—H26A	110.4	N7—C56—C55	107.9 (3)
C27—C26—H26B	110.4	H1WA—O1W—H1WB	102 (6)
C25—C26—H26B	110.4	H2WA—O2W—H2WB	108 (6)
			
C18—O3—N3—C16	−0.9 (4)	C26—C27—C28—N4	4.8 (5)
C46—O8—N8—C44	−0.3 (3)	O5—N5—C29—C34	159.1 (4)
O1—N1—C1—C2	3.3 (7)	O6—N5—C29—C34	−23.9 (5)
O2—N1—C1—C2	−179.0 (5)	O5—N5—C29—C30	−21.3 (6)
O1—N1—C1—C6	−178.1 (6)	O6—N5—C29—C30	155.7 (4)
O2—N1—C1—C6	−0.4 (7)	C34—C29—C30—C31	2.6 (5)
C6—C1—C2—C3	3.0 (7)	N5—C29—C30—C31	−176.9 (3)
N1—C1—C2—C3	−178.4 (4)	C29—C30—C31—C32	−1.6 (5)
C1—C2—C3—C4	−0.5 (6)	C30—C31—C32—C33	−1.1 (5)
C2—C3—C4—C5	−2.6 (6)	C30—C31—C32—C35	177.7 (3)
C2—C3—C4—C7	176.7 (3)	C31—C32—C33—C34	2.9 (6)
C3—C4—C5—C6	3.3 (7)	C35—C32—C33—C34	−175.9 (3)
C7—C4—C5—C6	−176.0 (4)	C30—C29—C34—C33	−0.9 (6)
C4—C5—C6—C1	−0.9 (8)	N5—C29—C34—C33	178.6 (4)
C2—C1—C6—C5	−2.3 (8)	C32—C33—C34—C29	−1.9 (6)
N1—C1—C6—C5	179.1 (5)	C39—N6—C35—C32	−167.4 (3)
C11—N2—C7—C4	−169.6 (3)	C39—N6—C35—C36	−42.9 (4)
C11—N2—C7—C8	−48.1 (4)	C33—C32—C35—N6	19.1 (4)
C3—C4—C7—N2	21.5 (5)	C31—C32—C35—N6	−159.7 (3)
C5—C4—C7—N2	−159.1 (4)	C33—C32—C35—C36	−102.7 (4)
C3—C4—C7—C8	−98.1 (4)	C31—C32—C35—C36	78.6 (4)
C5—C4—C7—C8	81.2 (4)	N6—C35—C36—C37	60.8 (3)
N2—C7—C8—C9	62.6 (3)	C32—C35—C36—C37	−175.3 (2)
C4—C7—C8—C9	−175.2 (3)	C56—N7—C37—C38	138.2 (3)
C28—N4—C9—C10	142.5 (3)	C53—N7—C37—C38	−55.9 (4)
C25—N4—C9—C10	−46.8 (4)	C56—N7—C37—C36	−96.9 (3)
C28—N4—C9—C8	−93.6 (3)	C53—N7—C37—C36	68.9 (4)
C25—N4—C9—C8	77.1 (4)	C35—C36—C37—N7	−174.8 (2)
C7—C8—C9—N4	−168.3 (3)	C35—C36—C37—C38	−49.4 (3)
C7—C8—C9—C10	−44.7 (4)	N7—C37—C38—C43	−37.9 (4)
N4—C9—C10—C15	−44.4 (4)	C36—C37—C38—C43	−161.7 (3)
C8—C9—C10—C15	−166.7 (3)	N7—C37—C38—C39	142.0 (3)
N4—C9—C10—C11	133.7 (3)	C36—C37—C38—C39	18.2 (4)
C8—C9—C10—C11	11.4 (4)	C35—N6—C39—C40	−169.0 (3)
C7—N2—C11—C12	−165.9 (3)	C35—N6—C39—C38	11.7 (5)
C7—N2—C11—C10	15.5 (4)	C43—C38—C39—N6	−178.4 (3)
C15—C10—C11—N2	−177.5 (3)	C37—C38—C39—N6	1.7 (4)
C9—C10—C11—N2	4.4 (4)	C43—C38—C39—C40	2.4 (4)
C15—C10—C11—C12	4.0 (4)	C37—C38—C39—C40	−177.5 (3)
C9—C10—C11—C12	−174.1 (3)	N6—C39—C40—C41	−179.5 (3)
N2—C11—C12—C13	−179.2 (3)	C38—C39—C40—C41	−0.2 (5)
C10—C11—C12—C13	−0.6 (4)	C39—C40—C41—C42	−1.6 (5)
C11—C12—C13—C14	−2.8 (5)	C40—C41—C42—C43	1.2 (5)
C12—C13—C14—C15	2.7 (5)	C41—C42—C43—C38	1.1 (5)
C13—C14—C15—C10	0.9 (5)	C41—C42—C43—C44	−174.8 (3)
C13—C14—C15—C16	−176.8 (3)	C39—C38—C43—C42	−2.8 (4)
C11—C10—C15—C14	−4.2 (4)	C37—C38—C43—C42	177.1 (3)
C9—C10—C15—C14	173.9 (3)	C39—C38—C43—C44	172.8 (3)
C11—C10—C15—C16	173.4 (3)	C37—C38—C43—C44	−7.3 (4)
C9—C10—C15—C16	−8.5 (4)	O8—N8—C44—C45	0.0 (4)
O3—N3—C16—C17	0.9 (4)	O8—N8—C44—C43	173.6 (3)
O3—N3—C16—C15	175.7 (3)	C42—C43—C44—N8	−54.3 (4)
C14—C15—C16—N3	−45.5 (4)	C38—C43—C44—N8	129.9 (3)
C10—C15—C16—N3	136.9 (3)	C42—C43—C44—C45	118.0 (4)
C14—C15—C16—C17	128.3 (3)	C38—C43—C44—C45	−57.8 (5)
C10—C15—C16—C17	−49.4 (4)	N8—C44—C45—C46	0.4 (4)
N3—C16—C17—C18	−0.6 (4)	C43—C44—C45—C46	−172.4 (3)
C15—C16—C17—C18	−174.7 (3)	C44—C45—C46—O8	−0.6 (4)
C16—C17—C18—O3	0.0 (3)	C44—C45—C46—C47	175.6 (4)
C16—C17—C18—C19	178.8 (3)	N8—O8—C46—C45	0.6 (4)
N3—O3—C18—C17	0.6 (4)	N8—O8—C46—C47	−176.3 (3)
N3—O3—C18—C19	−178.4 (3)	C45—C46—C47—C48	9.7 (6)
C17—C18—C19—C20	33.9 (5)	O8—C46—C47—C48	−174.3 (4)
O3—C18—C19—C20	−147.4 (3)	C45—C46—C47—C52	−167.0 (4)
C17—C18—C19—C24	−144.8 (4)	O8—C46—C47—C52	8.9 (5)
O3—C18—C19—C24	33.9 (4)	C52—C47—C48—C49	0.4 (7)
C24—C19—C20—C21	−0.7 (5)	C46—C47—C48—C49	−176.5 (4)
C18—C19—C20—C21	−179.4 (3)	C47—C48—C49—C50	0.5 (9)
C19—C20—C21—C22	−0.6 (6)	C48—C49—C50—C51	−0.4 (9)
C20—C21—C22—C23	1.0 (6)	C49—C50—C51—C52	−0.7 (8)
C21—C22—C23—C24	−0.1 (6)	C50—C51—C52—C47	1.7 (7)
C22—C23—C24—C19	−1.2 (6)	C48—C47—C52—C51	−1.5 (6)
C20—C19—C24—C23	1.6 (5)	C46—C47—C52—C51	175.3 (4)
C18—C19—C24—C23	−179.7 (3)	C56—N7—C53—C54	−20.9 (4)
C28—N4—C25—C26	−13.2 (4)	C37—N7—C53—C54	172.0 (3)
C9—N4—C25—C26	175.3 (3)	N7—C53—C54—C55	25.6 (4)
N4—C25—C26—C27	15.4 (5)	C53—C54—C55—C56	−22.0 (5)
C25—C26—C27—C28	−12.7 (5)	C53—N7—C56—O7	−172.8 (3)
C25—N4—C28—O4	−174.6 (3)	C37—N7—C56—O7	−5.7 (5)
C9—N4—C28—O4	−3.0 (5)	C53—N7—C56—C55	7.2 (4)
C25—N4—C28—C27	5.5 (4)	C37—N7—C56—C55	174.3 (3)
C9—N4—C28—C27	177.1 (3)	C54—C55—C56—O7	−170.2 (4)
C26—C27—C28—O4	−175.0 (4)	C54—C55—C56—N7	9.8 (4)

### Hydrogen-bond geometry (Å, °)

**Table:**

*D*—H···*A*	*D*—H	H···*A*	*D*···*A*	*D*—H···*A*
O1W—H1WA···O4^i^	0.83 (7)	2.07 (7)	2.904 (5)	173 (6)
O1W—H1WB···O4^ii^	1.03 (8)	1.87 (8)	2.877 (5)	167 (6)
O2W—H2WB···O7	0.97 (8)	1.80 (9)	2.754 (5)	165 (8)
N6—H6N···O2W^iii^	0.83 (4)	2.13 (4)	2.958 (5)	179 (5)
O2W—H2WA···O1W	0.80 (6)	2.09 (6)	2.883 (6)	175 (6)
C3—H3···N2	0.93	2.52	2.848 (6)	101
C9—H9···O4	0.98	2.50	2.857 (4)	101
C33—H33···N6	0.93	2.51	2.830 (6)	100
C37—H37···O7	0.98	2.49	2.872 (4)	103
C52—H52···O8	0.93	2.50	2.811 (5)	100

Symmetry codes: (i) *x*−1, *y*, *z*+1; (ii) −*x*+1, −*y*, −*z*+1; (iii) −*x*, −*y*+1, −*z*+1.
